# Medical ozone therapy reduces oxidative stress and testicular damage in an experimental model of testicular torsion in rats

**DOI:** 10.1590/S1677-5538.IBJU.2016.0546

**Published:** 2017

**Authors:** Mustafa Tusat, Ahmet Mentese, Selim Demir, Ahmet Alver, Mustafa Imamoglu

**Affiliations:** 1 Department of Pediatric Surgery, Kilis State Hospital, Kilis, Turkey; 2 Program of Medical Laboratory Techniques, Vocational School of Health Sciences, Karadeniz Technical, University, Trabzon, Turkey; 3 Department of Nutrition and Dietetics, Faculty of Health Sciences, Karadeniz Technical University, Trabzon, Turkey; 4 Department of Medical Biochemistry, Faculty of Medicine, Karadeniz Technical University, Trabzon, Turkey; 5 Department of Medical Biochemistry, Faculty of Medicine, Recep Tayyip Erdogan University, Rize, Turkey; 6 Department of Pediatric Surgery, Faculty of Medicine, Karadeniz Technical University, Trabzon, Turkey

**Keywords:** Ischemia, Oxidative Stress, Spermatic Cord Torsion

## Abstract

**Objective:**

Testicular torsion (TT) refers to rotation of the testis and twisting of the spermatic cord. TT results in ischemia-reperfusion (I/R) injury involving increased oxidative stress, inflammation and apoptosis, and can even lead to infertility. The aim of this study was to investigate the effect of ozone therapy on testicular damage due to I/R injury in an experimental torsion model.

**Materials and Methods:**

24 male Sprague-Dawley rats were divided into 3 groups; shamoperated, torsion/detorsion (T/D), and T/D+ozone. Ozone (1mg/kg) was injected intraperitoneally 120 minutes before detorsion and for the following 24h. Blood and tissue samples were collected at the end of 24h. Johnsen score, ischemia modified albumin (IMA), total antioxidant status (TAS), total oxidant status (TOS), and oxidative stress index (OSI) levels were determined.

**Results:**

Levels of IMA, TOS, OSI, and histopathological scores increased in the serum/tissue of the rats in the experimental T/D group. Serum IMA, TOS, and OSI levels and tissue histopathological scores were lower in the rats treated with ozone compared with the T/D group.

**Conclusion:**

Our study results suggest that ozone therapy may exhibit beneficial effects on both biochemical and histopathological findings. Clinical trials are now necessary to confirm this.

## INTRODUCTION

Testicular torsion (TT) results from the impairment of testicular and epididymal blood flow following rotation of the testicular spermatic cord and blood vessels (1). Progressive interruption of testicular venous flow then occurs. This subsequently leads to interstitial edema. Increasing and persistent edema halts arterial blood flow, thus resulting in parenchymal ischemia. Prolonged ischemia may conclude in organ loss. This is generally frequently seen in the newborn, children, and adolescents and can lead to acute scrotum and require emergency intervention (1, 2). Time intervals between torsion and detorsion and degree of spermatic cord torsion are the main factors determining the severity of testicular injury. In association with this loss of function, a decrease in fertility occurs in the ipsilateral testis, with testicular atrophy occuring in severe cases. Since blood flow in the testes is limited, these are particularly sensitive to ischemic injury (3). Although the basic pathological mechanisms are not yet fully understood, reactive oxygen species (ROS) resulting from ischemia and reperfusion (I/R) are known to play a role in tissue injury deriving from TT. I/R injury is characterized by neutrophil accumulation and increased pro-inflammatory cytokines, adhesion molecules, lipid peroxidation, and apoptosis (3-5). Oxidative phosphorylation is compromised due to insufficient oxygen caused by ischemia. Additionally, the Na^+^-K^+^ ATPase pump is inhibited as a result of an associated decrease in ATP levels. Intracellular Na^+^ and Ca^2+^ ion concentrations therefore increase. Intra and extracellular ion imbalance causes Ca^2+^ leakage into the mitochondria. An increase in mitochondrial Ca^2+^ activates various proteases and phospholipases, and cell lysis occur. These changes resulting from I/R injury trigger biochemical mechanisms, such as oxidative stress and inflammation (3). Various substances (phosphodiesterase inhibitors, vitamins, selenium, N-acetylcysteine, ethyl pyruvate, flavonoids, plant extracts, etc.) have been used in experimental studies to prevent this injury that can emerge following detorsion, and therefore the development of infertility (5-8). However, despite all the researches that have been performed, no additional therapeutic methods with easy clinical adaptation and proven utility have to date been obtained.

Medical ozone therapy is used in a wide spectrum for therapeutic purposes due to its antioxidant, anti-inflammatory, and antimicrobial effects. In contrast to treatment with pharmacological agents, ozone therapy provides defense against diseases by activating the body's antioxidant and anti-inflammatory pathways through the alarm reaction it creates, rather than through the classic drug-receptor relationship. The use of ozone therapy has been strongly emphasized in the treatment of diseases, such as chronic cutaneous ulcers, peritonitis, infected wounds, ischemic diseases, and joint problems (9, 10). In recent years in particular, studies have investigated the protective effect of ozone therapy against testis injury induced by various means. Aydos et al. demonstrated that intraperitoneal ozone therapy exhibits a protective effect against I/R-induced testicular injury in a rat TT model by reducing levels of apoptosis and oxidative stress (11). Recently, Salem et al. reported that ozone therapy exhibits protective effects against adriamycin-induced testicular toxicity in an experimental rat model by reducing levels of oxidative stress and nitric oxide (NO) (12).

The purpose of this study was to investigate the effects of medical ozone therapy on experimental testicular I/R injury in biochemical and histopathological terms using such traditional biochemical parameters as ischemia modified albumin (IMA), total antioxidant status (TAS), total oxidant status (TOS) and the oxidative stress index (OSI).

## MATERIALS AND METHODS

The experimental procedures in this research were approved by the Animal Care Ethical Committee of Karadeniz Technical University and were conducted in conformity with US National Institutes of Health guidelines. The experiments involved 24 male Sprague-Dawley rats (aged 4-6 months and with a mean weight of 250 g) fed on a standard chow pellet diet and with ad libitum access to tap water. These animals were housed in steel cages until the time of the study, under controlled lighting (lights on between 8:00 and 20:00h) at a temperature of 21-23°C. Water only was provided for the last 12h before the experiments.

Twenty-four rats were randomly assigned into one of three groups of eight members each. General anesthesia was induced with intramuscular injection of 10mg/kg of xylazine and 50mg/kg ketamine. The control group was subjected to a sham procedure (scrotal incision) only. In the other groups, the left testis was rotated 720 degrees clockwise to establish torsion. This was then maintained by fixing the testis. In the T/D group, detorsion was performed following 2 hours of torsion and then maintained for the subsequent 24 hours. These T/D procedures were repeated in the medical ozone group, but 1mg/kg ozone (Evozone BasicPlus, Germany), was administered intraperitoneally (IP) immediately prior to detorsion for 2 hours. Blood samples were collected from the abdominal aorta of all rats 24 hours after detorsion. Table-1 provides a summary of the procedures performed in the different experimental groups.

**Table 1:** A summary of the procedures in the experimental groups.

	Groups		
	Control	T/D	Medical Ozone Plus T/D
Torsion 0 min		+	+
Immediately before detorsion			1mg/kg ozone
Detorsion +2 hours		+	+
Blood and Tissue Samples +24 hours	+	+	+

**T/D =** Torsion/Detorsion

Blood specimens were placed into separetor tubes without anticoagulant and centrifuged at 2000xg for 10 min. The serum specimens obtained were divided into small volume tubes and stored at −80°C until biochemical measurements.

The colorimetric method described by Bar-Or et al. was used to determine IMA levels (13). The results were expressed as absorbance units (ABSU). Commercial colorimetric kits (Rel Assay Diagnostics, Gaziantep, Turkey) were used to determine TOS and TAS levels in rat sera. TOS results were expressed as μmoL H_2_O_2_ equivalent/L and TAS results as mmoL trolox equivalent/L. The TOS:TAS ratio was used as the OSI. For that purpose, the unit of TAS, mmoL trolox equivalent/L, was converted to μmoL trolox equivalent/L, and OSI was calculated using the formula:

OSI=[(TOS, μmoL H_2_O_2_ equivalent/L) / (TAS, μmoL trolox equivalent/L) x 10].

The testis tissue specimens obtained were fixed for 72h in Bouin's solution for histopatho- logical analysis. Care was taken to collect tissue specimens from approximately the same sections. The fixed tissue specimens were dehydrated by passing through 70%, 90%, 96% and 100% alcohol series. They were then rendered transparent by being passed through xylene solution. Following preparation of paraffin blocks, sections 5μm in thickness were taken using an automatic microtome. These were subjected to deparaffinization and then stained with hematoxylin-eosin (H&E). The preparates were analyzed under a light misroscope (Olympus BX 51, Tokyo, Japan). The Johnsen Testicular Biopsy Score system was used to evaluate testicular tissue injury. Under that system, testis tissues were evaluated semi-quantitatively in five different areas at high magnification (200x) under light microscopy (14). A pathologist evaluated the testicular tissues using standard light microscopy. This examination was completed in a random order and a blinded fashion. The histological sections were graded for testicular injury and spermatogenesis using the Johnsen score (JS). A minimum of 50 tubules were evaluated, with each tubule being scored from 1 to 10. Ten points expressed complete spermatogenesis with regular tubules; 9 points, many spermatozoa and irregular germinal epithelium; 8 points, presence of few spermatozoa; 7 points, no spermatozoa, many spermatids; 6 points, no spermatozoa, few spermatids; 5 points, no spermatozoa or spermatids; 4 points, few spermatocytes; 3 points, presence of spermatogonia; 2 points; sertoli cells only; and 1 point, complete absence of germ cells and spermatogenesis (6).

Statistical analysis was performed on SPSS 23.0 software. Kruskal-Wallis variance analysis (the Mann-Whitney U test with Bonferroni correction as post hoc) was used to compare the study groups. Statistical significance was set at p <0.05.

## RESULTS

Oxidative stress markers and histopatho-logical scores were the principal parameters for evaluating the degree of I/R damage and the effectiveness of medical ozone treatment in this study. No complication related to the T/D model or the administration of ozone therapy was observed. No mortality was observed in any group until the end of the experiment. Comparisons of group's biochemical parameters (IMA, TAS, TOS, and OSI) and histopathological scores are summarized in Table-2. Results are expressed as medians (interquartile range).

**Table 2:** A comparison of biochemical parameters and histopathological scores in the groups.

	Groups		
	Control	T/D	Medical Ozone Plus T/D
**IMA**	0.794 (0.783-0.806)	0.853^a^ (0.834-0.861)	0.737^b^ (0.710-0.778)
**TAS**	0.437 (0.421-0.472)	0.436 (0.300-0.478)	0.375 (0.357-0.389)
**TOS**	16.0 (13.7-18.8)	42.5^c^ (32.7-55.4)	18.0^b^ (15.3-21.5)
**OSI**	3.9 (3.0-4.4)	9.9^c^ (7.4-16.6)	4.9^b^ (3.6-6.4)
**HS**	10 (10-10)	5.20^c^ (4.90-5.35)	8.20^c^,^d^ (7.85-8.35)

Values are expressed as median (Percentiles 25-75). **IMA** = Ischemia Modified Albumin (Absorbance Unit: ABSU); **TAS** = Total Antioxidant Status (mmoL trolox equivalent/L); **TOS** = Total Oxidant Status μmoL H_2_O_2_ equivalent/L); **OSI** = Oxidative Stress Index; **HS** = Histopathological score; **T/D** = Torsion/Detorsion.

a **p=**0.006 compared with control group;

b **p=**0.0001 compared with T/D group;

c **p=**0.0001 compared with control group;

d **p=**0.001 compared with T/D group.

Serum IMA, TOS, and OSI levels were significantly higher in the T/D group compared to the control group (p=0.006, 0.0001, and 0.0001, respectively), but the levels of these parameters were significantly reduced by medical ozone therapy (p=0.0001 for all parameters). However, no significant difference was determined between the groups in terms of serum TAS levels (p >0.05).

**Figure 1:**
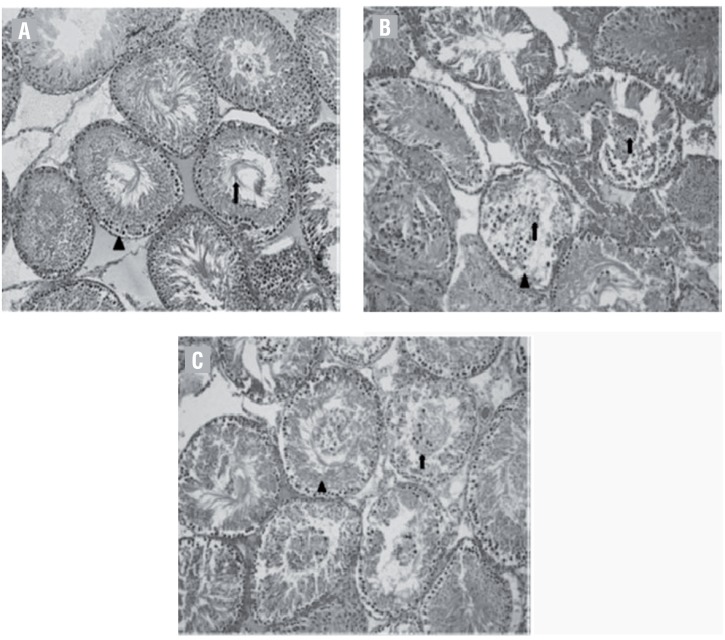
Ipsilateral testis (x200, hemotaxylin and eosin stain [H&E]). A) A section from the sham with group, normal seminiferous tubule epithelial structure (δ) and spermatozoons (↑) were observed. B) A section from the T/D group, seminiferous tubule germinal epithelial structure was completely poured (Δ), and luminal irregular germinal cells (↑) were observed. C) A section from the medical ozone therapy group, the germinal epithelial structure was regular (Δ) and regular lineage germinal epithelial cell (↑) observed in the lumen.

The histopathological score was significantly higher in the T/D group compared to the control and medical ozone therapy groups (p=0.0001, and 0.001, respectively). The histopathological score in the medical ozone therapy group was also significantly lower compared to the score in the T/D group (p=0.001). In the control group, regular seminiferous tubular morphology with normal spermatogenesis were detected. In the T/D group, seminiferous tubule germinal epithelial structure was completely poured. Spermatozoa were not available in the lumen and vasoconstriction was partly observed in the intertubular area. The seminiferous tubule epithelial structure was more regularly in the medical ozone therapy group compared to T/D group. In the medical ozone therapy group, germinal epithelial cells showed regular alignment in the lumen and spermatozoa were partly observed (Figure-1).

## DISCUSSION

Testicular torsion is one of the emergency conditions frequently seen in the newborn and adolescent periods, and one that can lead to testicular injury or even subfertility. Since the testis is one of the most sensitive organs to hypoxia, even short-term torsion may lead to significant injury, such as hypoxia in testicular tissue, cell damage, and cell death. Oxidative stress and the inflammatory process associated with ROS are involved in the etiology of I/R injury observed during TT-detorsion. Irrespective of the etiological factor and despite research into alternative medical treatment models, emergency surgical intervention remains a valid and the most commonly applied treatment modality (7). Many pharmacological agents, such as phosphodiesterase inhibitors, vitamin C and E, selenium, flavonoids, NSAID, ethyl pyruvate, and N-acetyl cysteine have been investigated in animal models for their potential as adjunctive therapies to the surgical repair of TT. These chemicals generally have anti-inflammatory, antioxidant or ROS-scavenging properties (5-8). However, these chemical agents are little employed in routine clinical practice for reasons, such as insufficient effectiveness, safety concerns, and a lack of information concerning dosages (7). In recent years in particular, ozone therapy has been shown to exhibit positive effects on wound healing and pathological conditions, such as age-related macular degeneration and ischemic and infectious diseases. These effects of medical ozone therapy have been attributed to more than one mechanism (such as increasing 2, 3-bisphosphoglycerate levels in erythrocytes, providing platelet activation, and raising antioxidant enzyme levels) (9, 10). The purpose of this study was therefore to determine the protective effect of medical ozone therapy against I/R injury induced in the rat testis using oxidative stress markers and histopathological scoring. The measurement of changes in IMA, TAS, TOS, and OSI is often used as an index of oxidative stress in biological systems (15). These markers were therefore employed to evaluate oxidative stress in this study. Our results show that medical ozone therapy significantly reduced IMA, TOS, and OSI values that normally rise in a TT model. Histopathological analysis also revealed that medical ozone therapy significantly reduced scores that increase as a result of torsion.

Previous studies have also investigated the protective effect against testicular injury of medical ozone therapy. Ekici et al. reported that ozone therapy protected against I/R damage in an experimental unilateral TT model in rats. Ozone therapy significantly suppresses and induces malondialdehyde (MDA) and glutathione (GSH) levels, respectively. It has also been shown to significantly protect testicular tissue against I/R injury measured on the basis of Johnsen scores (16). Aydos et al. determined that medical ozone therapy exhibited a protective effect against TT-induced I/R injury by reducing apoptosis and iNOS and increasing catalase enzyme activity (11). Salem et al. recently evaluated the protective effect of ozone treatment on adriamycin-induced testicular toxicity. They showed that medical ozone therapy exhibited positive effects on sperm numbers, motility, and viability in an induced model of testis injury. That study also reported that medical ozone therapy suppressed oxidative stress by reducing MDA and NO levels (12).

Recent studies have shown that ozone pre-conditioning is an effective means of preventing I/R damage in various organs, such as the liver, lung, intestine, ovary, and kidney. Chen et al. demonstrated that ozone therapy inhibits inflammation and apoptosis after renal ischemia/reperfusion injury in rats. They observed that increased levels of oxidative stress and inflammation (myeloperoxidase activity and the expression of interleukin-1 beta, tumor necrosis factor alpha, and intercellular adhesion molecule-1) markers were reduced by ozone therapy (17). Di Filippo et al. reported that acute oxygen-ozone therapy protects rats against the I/R damage in an experimental acute myocardial infarction model. Infarct size and levels of 3-nitrotyrosine (a product of protein oxidation), interleukin-6, interleukin-8, and caspase 3 are reduced by medical ozone therapy in a concentration-dependent manner (18). Haj et al. observed that treatment of I/R rats with ozone/oxygen mixture resulted in a significant decrease in intestinal injury scores and numbers of apoptotic cells in the ileum (19). Onal et al. reported that ozone administration increased the levels of superoxide dismutase (SOD), glutathione peroxidase (GPx), catalase (CAT), and TAS and reduced the level of TOS in an experimental intestinal I/R model. No difference was observed between the groups in terms of MDA or protein carbonyl levels in that study. Histopatho-logical evaluation showed that pre-treatment with peritoneal ozone prevented intestinal mucosal injury caused by I/R (20). Sayar et al. recently reported that medical ozone therapy exhibits a protective effect on rat ovaries in an I/R injury model by reducing oxidative stress (21).

ROS derive from normal metabolic reactions and are involved in a wide range of processes, including apoptosis and cell signaling. They also oxidize lipids contained in the cell and mitochondrial membranes, thus modifying membrane permeability and compromising cellular integrity. Ozone therapy is associated with effective regulation of oxidative stress at the cellular level. Previous studies have identified numerous benefical biochemical effects of ozone therapy that raise antioxidant activity, which is believed to ready tissues for exposure to oxidative stress. The pathophysiology of the anti-inflammatory and antioxidant characteristics of ozone administered at therapeutic doses is still unclear, since ozone decomposes numerous components of blood. Ozone has been reported to increase the activity of antioxidant enzymes, such as GPx, SOD, and CAT. These enzymes ready the host for ROS-induced physiopathological conditions (2, 18). In the present study, serum IMA, TOS and OSI levels increased in untreated rats but, decreased in those administered ozone therapy. This suggests that one potential benefical effect of ozone may be to minimize tissue damage via improved antioxidant enzyme activity.

Ozone therapy may prevent injury if oxidant status is dominant. However, if there is no challenge to the oxidant/antioxidant balance, then ozone may be deleterious. In order to obtain maximum benefit from the biological effects, the dose of ozone applied should be calculated very carefully (22). The concentration of ozone in medical therapy in previous reports varies between 0.1 and 4mg/kg, and it was administered IP. Both concentration of ozone (1mg/kg) and treatment time (2h) in this study were therefore compatible with previous studies (2, 11, 16, 18, 22).

## CONCLUSIONS

Our data suggest that ozone therapy reduces the severity of I/R injury in an experimental model of TT by inhibiting oxidative stress. Our findings indicate that outcomes of TT can be improved be employing ozone therapy as an adjuvant therapy. However, further studies involving well-designed experimental models are now needed to clarify the mechanisms of action by which ozone exerts its effects.
